# A model for understanding teachers’ intentions to remain in STEM education

**DOI:** 10.1186/s40594-017-0061-8

**Published:** 2017-04-05

**Authors:** John R. McConnell

**Affiliations:** Department of Educational Specialties, Austin Peay State University, P.O. Box 4545, Clarksville, TN 37044 USA

**Keywords:** Math and science teachers, Satisfaction with salary, Structural equation modeling, Teacher retention

## Abstract

**Background:**

This study examined the relationships of various teacher retention factors with the intentions of math and science teachers to remain in the profession. With data collected from the 2007–08 Schools and Staffing Survey, a sample of 6588 secondary math and science teachers across public schools in the USA was used for structural equation modeling.

**Results:**

Socioeconomic impact, student truancy, and years of experience all showed direct relationships with teacher autonomy, while administrative support, teacher autonomy, and satisfaction with salary were all directly related to these teachers’ intentions to remain in the profession. Of these teacher retention factors, satisfaction with salary was found to have the strongest relationship.

**Conclusions:**

By understanding what factors are associated with the intentions of math and science teachers to continue teaching, educational policymakers and practitioners will have practical guidance in helping them make decisions to improve the retention of these teachers in secondary public schools, on whom the fields in STEM are so dependent.

## Background

There is a shortage of high-quality educators in the USA, and an effort to understand the effects on teacher recruitment and retention is currently underway at federal and state levels of governance (e.g., H.R. 1161, 2009). Although more teacher certificates are being awarded nationally, states like Minnesota, New York, Oklahoma, Virginia, and Washington are witnessing drops in new teacher licenses by one third to one half in the last 4 years (Title II HEA 2015). States without this problem, like South Dakota, still struggle to meet demand due to drastic increases in student enrollment (South Dakota Department of Education 2015). The lack of certified teachers is more prevalent in certain fields, with states finding difficulty filling math and science positions, hiring special education and bilingual teachers, and staffing their middle and high schools (Cowan et al. 2015). In areas with high poverty and minority populations, these problems are even more acute (NCES 2015).

For states that do not have a shortage, students may still be taught by teachers out of their fields. For instance, in 2012, 43% of high school biology teachers reported they were teaching classes out of their field, with 12% stating that less than half of their classes were related to biology (NCES 2015). A report by the President’s Council of Advisors on Science and Technology (2012) predicted a shortage of one million college graduates in those areas over the next decade, so recruitment and retention of these individuals in the teaching profession will only become more tenuous. Because of this strain on teacher ranks, especially with regards to highly effective teachers, there are concerns about the quality of teachers in the USA and the subsequent effect it has on the education of its schoolchildren (Murnane et al. 1991). This is particularly true in the areas of math and science (Henke et al. 2001; Ingersoll 2001), where the retention of individuals skilled in those areas is seen as especially beneficial to student learning.

Not only do they affect students and their education, teacher retention policies impact the educational institutions and the educators who can work or are working within them. Low retention rates can bode poorly for teacher recruitment, increase overhead expenditures for school districts, and impinge upon program continuity and planning. Most importantly, these policies affect school districts by guiding the way they hire teachers and maintain their ranks. For prospective and current teachers, including those in the areas of math and science, these policies will influence their decisions to enter and remain in the profession, respectively. To help shape these policies, an improved understanding of what affects the retention of these teachers in public schools is required.

### Statement of the problem

Clearly, the national problem of staffing schools with highly effective math and science teachers is far from uniform and requires differentiated policy solutions. For instance, urban school districts tend to report larger class sizes and more problems with student truancy, yet typically provide higher wages (Jacob 2007). Rural schools districts, on the other hand, tend to offer smaller class sizes with fewer problems with discipline but furnish less compensation for their teachers (Monk 2007). Compared to their suburban counterparts, both serve relatively more students from disadvantaged neighborhoods with tighter budgets (Jacob 2007; Monk 2007). In addition to the types of communities they serve, it is important to consider the geographical, historical, and political contexts of the teacher shortage problem. The merging of school districts in financially constrained metropolitan areas and consolidation of individual schools in less populated areas of the country have diminished the number of school districts of the years, creating increased administrative burdens. All of these factors affect the recruitment and retention of highly effective teachers, including those skilled in math and science. How should educational policymakers address these shortages in such a way that is tailored to their individual constituencies?

There are economic and sociological theories at play in understanding why math and science teachers remain in or leave the profession. The economic theory of supply and demand influences how well school districts can fill and maintain their ranks of math and science teachers by shaping their career decisions about education and the alternative professional opportunities available to them (Guarino et al. 2004). This well-established theory applies to both the employers, i.e., districts and schools, and the employees, i.e., math and science teachers (Guarino et al. 2004). From the viewpoint of districts and schools, these economic forces influence how well they can fill and maintain their ranks of math and science teachers. From the viewpoint of math and science teachers, these same forces help shape their decisions as to whether they should enter or remain in the classroom. Sociological theories of organizational structure and management apply to the working conditions individuals skilled in math and science find themselves in when working as public school teachers and, when compared to those perceived in other fields, help to influence the satisfaction they have with and the decisions they make about their career paths (Newton et al. 2012). The working conditions in a school, as in any workplace, contribute to whether its teachers decide to stay or leave that school, and these conditions are manifested at the school level by school, district, state, and federal policies (Newton et al. 2012). Taken together, these economic and sociological principles provide a logical framework to help guide the investigation, understanding, and evaluation of how math and science teachers are retained in public schools.

### Economic forces in the labor market

Supply and demand in the labor market is a basic theory in economics. In the labor market for teachers, supply refers to the number of teachers willing to teach at a given level of compensation, and demand refers to the number of teaching positions open at a given level of compensation. Compensation includes both pecuniary and non-pecuniary benefits, with the former including benefits like salary, health coverage, and a pension and the latter including benefits like preferential scheduling, favorable working conditions, and a sense of altruism. Under the economic laws of supply and demand, an undersupply of teachers, an increase in the demand for teachers, or both simultaneously, can result in a teacher shortage, and vice versa. Changes to local, state, and federal policies in regards to teacher staffing are, in turn, driven by these fluctuations in supply and demand to the extent of how much one outstrips the other.

Of particular concern during a teacher shortage is the likelihood for policies to be changed to lower hiring standards in order to fill the number of open teaching positions. This can lead to an increase in the number of under- and non-qualified teachers in the field and, tragically, a decrease in student performance and achievement. Over generations, this can have a compounding effect as fewer and fewer students gain competency in their subject areas, thus further reducing the number of qualified individuals in the workforce. Some researchers have suggested that this phenomenon is more pronounced in some teaching fields more than others, with math and science being particularly prone to the compounding effect of teacher shortages (Grissmer and Kirby 1992, 1997; Liu et al. 2008; Murnane et al. 1991; Weiss and Boyd 1990).

Unsurprisingly, the shortage of math and science teachers in public education is precipitously high. Concerns over the lack of math and science teachers started to arise 30 years ago with calls by national organizations like the US Department of Education and the National Academy of Sciences publicizing the increasing severity of this problem and the importance of resolving the threat it poses to the educational quality, economic well-being, and national security of this country. The National Science Board (2012) reports that the number of workers in fields involving math and science grew from about 182,000 in 1950 to 5.4 million in 2009, and this represented an average annual growth rate of 5.9%, which was much greater than the 1.2% growth rate for the total workforce during this period. As the number of students entering the burgeoning fields of science, technology, engineering, and math (STEM) continues to swell, the need for qualified teachers in these fields will also intensify.

Educational institutions have responded to this growing need for math and science teachers with policies that promote their recruitment and retention. These policies have traditionally targeted pecuniary benefits in order to increase compensation. Monetary incentives, such as signing bonuses, student loan forgiveness, housing assistance, and tuition reimbursement, have all been established to spur recruitment and retention (Hirsch et al. 2001; Feistritzer 1997; Liu et al. 2008; Rice et al. 2008). Additional policies have focused on improving working conditions and job satisfaction by implementing programs that encourage mentoring, professional development, career advancement, and the elevated prestige of the teaching profession.

### Sociological conditions in the school

While an underlying economic framework is important to the problem of teacher retention and many studies have relied on this perspective, there is another critical aspect of the issue: the sociological context in which teachers work. A basic tenet of organizational management theory states that the organizational and occupational contexts in which individuals work will affect their satisfaction with the organization and the decisions they make as to whether they choose to remain a part of or leave it. Applied to math and science education, working conditions for teachers in these areas may hold sway in their decisions to remain at or leave their schools. These working conditions include but are not limited to high levels of student misbehavior, low workplace safety, lack of administrative support, scarce classroom resources, low faculty input into school decision-making, and inadequate opportunity for professional development.

From a sociological perspective, differences between schools are especially pronounced in relation to the retention of math and science teachers. In particular, disadvantaged public schools have among the highest rates of math and science teacher attrition. Economic considerations do not appear to be solely responsible for the high rates of math and science teacher turnover in these schools; they are also attributable to unfavorable job conditions. Resolving the organizational and occupational challenges facing math and science teachers in the classroom is not easy, but adjusting some of these working conditions may be less expensive than other organizational reforms and are necessary in retaining these teachers in public school systems.

In addition to improving the working conditions of teachers, these sociological considerations can also elevate the prestige of the occupation. Unlike in many European and Asian countries, the power and prestige of teaching in secondary education in the USA is poor (Etzioni 1969; Lortie 1975; Tyack 1974). Organizational structure and how members of an occupation are treated and managed are inextricably tied to occupational status (Abbott 1988; Freidson 1986). Despite the highly skilled and demanding work required of math and science teachers, their standing in society is incommensurate with their educational and work requirements, and promoting educational policies that upgrade the social status of the occupation will help alleviate the problem with their retention.

### Teacher and school characteristics

Given these theoretical considerations, various teacher retention factors, including both teacher and school characteristics, have come to light as being intimately tied to teacher job satisfaction. Some teacher characteristics identified as reliable indicators of their job satisfaction include salary (Bloland and Selby 1980; Bobbitt et al. 1991; Hanushek and Rivkin 2007), teacher autonomy (Billingsley 1993, 2003; Jones et al. 2003), and years of experience (Hanushek et al. 2004; Ingersoll 2001). Some school characteristics deemed as influential to teacher job satisfaction include administrative support (Ingersoll 2001; Johnson and Birkeland 2003), the socioeconomic impact of student families (Hanushek et al. 2004), and student truancy (Friedman 2000). These teacher and school characteristics served as the variables used in this study.

#### Satisfaction with salary

Empirical evidence establishing the association between salary and teacher retention indicate that higher salaries tend to correlate with higher teacher retention rates (Brewer 1996; Gritz and Theobald 1996; Ladd 2007; Mont and Rees 1996). In particular, salary differentials between the teaching profession and those in other non-profit and for-profit sectors have a more pronounced effect on teacher shortages in math and science, as individuals with technical skills in math and science are presented with more career alternatives relative to those who do not (Ladd 2007; Levin 1985). Even researchers investigating non-pecuniary factors impacting teacher retention like working conditions and the intrinsic benefits teachers gain in the field, e.g., imparting knowledge and life skills on young people for the betterment of society, acknowledge that salary remains an important factor in teacher retention (Hall et al. 1992; Hounshell and Griffin 1989; King 1993; Podgursky et al. 2004).

While it appears from the literature that higher salaries result in greater teacher retention, there is a lack of research that addresses whether or not teachers are satisfied with their salaries and what bearing their satisfaction has on their decisions to remain in the teaching profession. This distinction between salary and satisfaction with salary is a subtle yet potentially significant one as an individual with a relatively low salary may still be satisfied with it just like someone with a relatively high salary may never be satisfied with it. Instead of looking at absolute salary levels, relative levels in satisfaction with salary were employed in this study. In the targeted fields of math and science, where, for the same educational requirements, more alternative avenues for employment exist (and often with higher pay), educational policies concerning teacher compensation should probably take into account the relative level of teacher satisfaction with salary to those in competing fields.

#### Teacher autonomy

Teacher autonomy is described as the teachers’ “need to have a measure of control over their actions and have input into decisions that affect their jobs (Jones et al. 2003, p. 140). Whereas prior research concentrated on how teacher autonomy is associated with student factors such as achievement and motivation (Caprara et al. 2006), more current research turned attention toward the association of teacher autonomy with their job satisfaction (Klassen and Chiu 2010), reinforcing that teacher autonomy is an important contributor to whether they decide to remain in the profession (Billingsley 1993, 2003).

#### Years of experience

In terms of teacher experience, studies show that teacher retention rates are precipitously low for newly hired teachers (Grissmer and Kirby 1992; Hanushek and Rivkin 2006; Kirby et al. 1999; Murnane et al. 1991; Stinebrickner 1999). Research also indicates that teachers with more years of experience tend to perceive greater teacher autonomy until they near retirement age (Hanushek and Rivkin 2006; Ingersoll 2001).

#### Administrative support

The impact of administrative support on teacher working conditions and teacher retention is undeniable. Administrative support encompasses a wide range of school-level policies, affecting school concerns such as student discipline, staff morale, teacher resources, school culture, and communication and collaboration between school personnel. Research consistently indicates that having more types of support and providing that support more extensively to teachers on the job lowers the likelihood that they will leave their jobs (Ingersoll 2001; Johnson and Birkeland 2003; Smith and Ingersoll 2004; Weiss 1999). Hounshell and Griffin (1989), in fact, found that science teachers with low job satisfaction attributed their frustration to problems with student discipline, excessive time commitments as determined by administrators, and high workload.

#### Socioeconomic impact of student families

Another extrinsic school characteristic that factors into the working conditions of teachers and thus their retention is the demographic makeup of the student population they serve. Schools with higher proportions of low income and minority students incur lower teacher retention rates than schools with higher proportions of high income and non-minority students (Boyd et al. 2005; Carroll et al. 2000; Hanushek et al. 2004; Scafidi et al. 2007; Shen 1997; Smith and Ingersoll 2004). Students from low income families experience poor student health due to less accessibility to proper healthcare providers, lack of parental involvement due to a lack of parent(s) or parental apathy, and less material, emotional, and psychological supports due to a dearth of resources at home. The research suggests that schools serving student populations in high-poverty communities have a greater challenge in retaining the teachers they need to end or restrict the promulgating problem that poverty presents; in turn, this reduces the control teachers believe they can have on student outcomes, i.e., teacher autonomy (Hanushek et al. 2004; Shen 1997). With math and science teachers more likely to leave than other teachers, their retention poses a particularly exacerbating problem for schools to address (Henke et al. 2001; Ingersoll 2001).

#### Student truancy

Research shows that student truancy also influences the autonomy that teachers think they have with their work. Truancy refers to the intentional absence from school that is not authorized by the school. For the purposes of this study, truancy and any teacher perceptions of problems with truancy pertain to students skipping class, tardiness, absenteeism, and students dropping out of school. Because truancy reduces the amount of time teachers spend with students, thereby hindering their ability to affect student outcomes, it can cause teachers to feel less in control (Friedman 2000) and experience more job stress (Friedman 2000; Furlong et al. 1994). Over time, this reduced self-efficacy and elevated job stress can impact their overall job satisfaction and bring on a desire to leave the teaching profession (Borg and Riding 1991; Byrne 1994; Hastings and Bham 2003; Luekens et al. 2004).

### Teachers’ intentions to remain in STEM education

As previously discussed, many studies on this topic tie teacher retention to job satisfaction in an attempt to study teacher retention using a continuous variable. Teacher retention remains distinct from teacher job satisfaction, however, as teachers may remain at their jobs for reasons other than being satisfied with their jobs. These reasons may include satisfaction with their salary, the teaching autonomy they possess, years of experience, and low levels of various job stressors. Conversely, other teachers may leave their teaching positions despite being satisfied with their jobs. These reasons may include certain life events, exposure to a more appealing career opportunity, and retirement. Because of this distinction, this study chose to examine teachers’ intentions to remain in the profession instead of their job satisfaction as a proxy for teacher retention. Not only would this reflect what teachers want to do as a result of factors related to them and their schools, but it would still maintain the continuous nature of the data for analytical purposes.

Recent studies pioneered the use of sufficiently sophisticated methodologies to examine this multivariate issue of teacher retention. Studies by Klassen et al. (2009, 2010), in particular, employed the use of structural equation modeling (SEM) to investigate the relationships of various factors with job satisfaction and teacher retention with teachers in Canada. However, SEM using data indigenous to the USA is needed to generalize findings to the USA. The theoretical constructs of these variables must also be agreed upon and reinforced by other experts in the field if researchers are to build upon established models or offer alternative models for practical use. Moreover, having additional models to explain the data would help formulate the most plausible substantive explanation of what promotes teacher retention. No research to date has been conducted utilizing SEM to evaluate the retention of math and science teachers in secondary public schools in the USA. Methodologically, this study extended the work of prior research by using a large, nationally representative data set indigenous to the USA to test a model that depicts its retention of math and science teachers in secondary public schools, where student interests in science, technology, engineering, and mathematics (STEM) are usually formed.

To better understand the factors related to the retention of math and science teachers in secondary public schools, this study aimed to assess the relative importance of various school and teacher characteristics to their intentions to remain in the profession. In light of the economic and sociological theories related to the retention of math and science teachers in secondary public education, all of these factors were deemed receptive to education policy on the federal, state, and district levels.

**Fig. 1:**
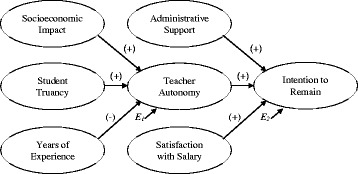
Model of hypothetical relationships in the study

This study tested a hypothetical model for the retention of math and science teachers in secondary public schools that accounts for these teacher and school characteristics and their relationships as described in the literature. Two research questions were addressed. First, how are the socioeconomic impact of student families, student truancy, and years of teaching experience related to teacher autonomy? Second, how are administrative support, teacher autonomy, and satisfaction with salary related to teachers’ intentions to remain in the profession? A hypothetical model was formulated for this study and is represented in Fig. 1, where *E*_*i*_ signifies the error term.

## Methods

### Origin of the data

The Schools and Staffing Survey originated as a response to the teacher shortage in the USA that began to emerge in the 1980s and was developed to collect data about schools, administrators, teachers, and teaching staff. It provides researchers and policymakers a uniquely valuable data set from which to study teaching and schooling. Because public school teachers of math and science were the focus of this study, only the public school questionnaire of the Schools and Staffing Survey 2007–08 (SASS08) was used. This questionnaire sought to obtain information about public school teacher characteristics, general conditions in public schools, these teachers' perceptions of school climate and problems, teacher compensation, and basic characteristics of their student populations. This data set was chosen because it provided the most relevant and recent data on the variables of interest in this study.

Data collection for SASS08 was carried out by the US Census Bureau using a mail-based questionnaire, followed up with telephone and field interviews for teachers who failed to return their questionnaires. Each questionnaire was then coded and checked for missing data. Four sources for data imputation were used for determining values for unanswered questionnaire items: other items on the questionnaire, related components of SASS08, the 2005–2006 Common Core of Data school survey, and records for sample cases with similar characteristics (US Department of Education 2008).

### Participants

The questionnaire elicited survey responses from 38,240 public school teachers, and these were used to compile the data set employed in this study. From this data set, only regular full-time teachers were selected, and all part-time, itinerant, and substitute teachers were excluded. Then, the sample was parsed to include only secondary public school teachers who taught subjects related to math and science in grades 6 through 12 (*N* = 6,588). Their demographic characteristics are presented in Table 1.

**Table 1:** Demographic characteristics of the participants

Demographic	Frequency	Percentage
Gender		
Male	2873	43.6
Female	3715	56.4
Race		
White	5936	90.1
Black	403	6.1
Asian	165	2.5
Native Hawaiian or Pacific Islander	32	0.5
American Indian or Alaska Native	133	2.0
Hispanic origin	200	3.0
Grade levels		
6^th^	523	7.9
7^th^	1337	20.3
8^th^	1470	22.3
9^th^	3641	55.3
10^th^	4318	65.5
11^th^	4354	66.1
12^th^	4150	63.0
Ungraded	262	4.0

*Note*. Because participants may identify with more than one ethnicity (e.g., White and Hispanic), the percentages for race did not add up to 100%. The same was true for grade levels, as many secondary school teachers teach more than one grade level in the same year

Of the participants in the sample, 54.2% were math teachers and 64.8% were science teachers, with 25% teaching in grades 6 through 8 and 75% teaching in grades 9 through 12.

### Procedure

The preparation of the data included a preliminary analysis, recoding of the data, the selection of items to be used in the modeling, weighting of the data, and both exploratory and confirmatory factor analyses.

#### Preliminary analysis and recoding of variables

A preliminary analysis was first conducted to inspect the data for completeness and accuracy and to help determine which questionnaire items to use to construct the latent variables for the study. Pre-selected potential items for use were first checked for possible outliers that may affect the goodness of fit of the model, using bivariate scatter plots and histograms. Following the preliminary analysis, the original coding of the data was reviewed to find questions with wording that reversed their direction in respect to the other questions, which could jeopardize parameter estimation and model fit. For example, one question (T0286) asked the extent to which “the school administrator’s behavior toward the staff is supportive and encouraging” and another question (T0301) asked the extent to which “the amount of student tardiness and class cutting in this school interferes with my teaching,” with response choices ranging from strongly agree (numbered 1) to strongly disagree (numbered 4). A lower value in the former item would indicate a favorable condition, while the same would indicate an unfavorable condition in the latter question. Therefore, items were recoded in such a way that a lower value for an answer would consistently indicate a less favorable condition and a higher value for an answer would indicate a more favorable condition (see Table 7 in Appendix 1). In the event that there was a case with missing data, it was deleted as the sample size was shaping to be sufficiently large for the study.

#### Selection of items

Prior to any modeling that was performed, items relating to each of the variables of interest in this study were selected from the survey based on the theoretical and methodological considerations from prior research. While the operationalization of the variables may change based on the results of factor analyses, all possible items for each of the variables were considered for inclusion. Table 7 in Appendix 1 contains all of the specific questionnaire items from SASS08 that were considered for use in this study, along with information on their scaling and recoding.

##### Teacher level factors

Three teacher level factors were explored in this study, with all of them serving as independent (exogenous) variables with respect to the intentions of math and science teachers to remain in the profession. These factors were years of experience, teacher autonomy, and satisfaction with salary.

Years of experience was an exogenous variable in this study that utilized a single questionnaire item asking the teacher the number of years he or she has worked as a full-time elementary or secondary teacher in a public school.

Teacher autonomy served as a locally endogenous variable in this study and as an exogenous variable to math and science teachers’ intentions to remain in the profession. Six questionnaire items from SASS08 were considered for use in measuring this variable, using Klassen and Chiu’s study of 2010 as a basis for their selection. In their study, they had three teacher autonomy factors specific to classroom management, student engagement, and instructional strategies and included survey questions like, “How much can you do to control disruptive behavior in the classroom?”, “How much can you do to implement a variety of assessment strategies?”, and “How much can you do to implement alternative strategies in your classroom?” (Klassen and Chiu 2010). For this study, a single teacher autonomy variable was used with the consideration of six questionnaire items from SASS08 to be included in its scale. These items referred to the teacher’s perception of his or her ability to effectively control the following areas of their craft: (1) selecting textbooks and other instructional materials, (2) selecting content, topics, and skills to be taught, (3) selecting teaching techniques, (4) evaluating and grading students, (5) disciplining students, and (6) determining the amount of homework to be assigned. Each of these items was based on a 4-point Likert scale with 1 = no control and 4 = a great deal of control.

Satisfaction with salary was an exogenous variable in this study specifically referring to, not the actual value of the teacher’s annual base salary, but the teacher’s satisfaction with his or her salary in relation to what is required of him or her to earn it. This satisfaction can be influenced by other factors besides the actual yearly dollar amount that a teacher makes from work as a teacher, like the amount and difficulty of the commensurate work required to earn that money, the degree of perceived support for professional development, the academic qualifications and teaching capabilities a teacher possesses, etc. Two questionnaire items from SASS08 were deemed relevant in the consideration of how to measure teachers’ satisfaction with their salaries. The first question asked to what extent the teacher was satisfied with his or her teaching salary and the second question asked to what extent the teacher would leave teaching if he or she could get a higher paying job. Both questions used a 4-point Likert scale with 1 = strongly agree and 4 = strongly disagree. The first item was reversely recoded for interpretability.

##### School level factors

Factors related to the sociological conditions in the school were also treated as independent (exogenous) variables in this study. Initially, it was not known what specific underlying factors existed or how these factors would be operationalized in regards to the sociological or working conditions in which teachers found themselves at their schools. Klassen and Chiu’s study of 2010 used job stress as this factor in their model, but, after examining the number of items related to job stress in the SASS08 survey, it was evident that factor analyses may be needed to pare down the number of relevant items and/or differentiate a number of factors that underlie the sociological aspects of a teacher’s decision to remain on the job. Theoretically, it was known that this construct in the study would comprise of the non-pecuniary elements of a teacher’s job that he or she perceives as either making it more amenable or difficult. As discussed earlier, these can include but are not limited to administrative support, student behavior, parental support, opportunity for professional development, and other environmental factors. This study chose to concentrate on those factors most open to policy intervention; accordingly, those items most closely related to these factors were considered for inclusion in this study.

SASS08 asked teachers a series of questions about their schools’ climates and working conditions, including their interactions with administrators and colleagues, job safety and student behavior, parental support and involvement, teaching resources and development, duties and paperwork, teacher recognition, truancy and absenteeism, student poverty and health access, and their attitudes toward various problems commonly encountered in public school systems. Of these items, 32 were chosen for possible inclusion in this study.

First, a series of 16 questions asked the teacher the extent to which he or she agreed with a number of statements about his or her principal and administration, fellow teachers, students, and school conditions. All these items used a 4-point Likert scale ranging from 1 = strongly agree to 4 = strongly disagree. These included some questions regarding administrative support, which Ingersoll (2001) deemed pertinent to the study of teacher retention. For example, “the school administration’s behavior toward the staff is supportive and encouraging,” “my principal enforces school rules for student conduct and backs me up when I need it,” and “the principal knows what kind of school he or she wants and has communicated it to the staff” were included as items in the survey.

Second, in regards to student behavior and socioeconomic influences, there were 10 questions asking the teacher the extent to which student tardiness, student absenteeism, student class cutting, student apathy, lack of parental involvement, poverty, and poor student health were a problem at his or her school. These items also had a 4-point Likert scale with possible response options ranging from 1 = serious problem to 4 = not a problem.

Third, there were six items that asked teachers the extent to which they agreed with the following statements about their attitude toward the school in which they worked, like “The stress and disappointments involved in teaching at this school aren’t really worth it” and “I like the way things are run at this school.” These items were also based on a 4-point Likert scale with 1 = strongly agree and 4 = strongly disagree. Items with reverse direction from the others were reversely recoded for interpretability. The identification of underlying factors and the selection of the exact items to be used for those factors were based on the results of exploratory factor analysis.

##### Teachers’ intentions to remain in the profession

The teachers’ intentions to remain in the profession was a dependent (endogenous) variable in this study referring to the teachers’ possible decisions to keep teaching. To measure the construct of a teacher’s intention to remain in the profession, this study considered two questionnaire items. The first asked how long the teacher planned on remaining in teaching, with five possible response options: (1) as long as I am able, (2) until I am eligible for retirement, (3) will probably continue unless something better comes along, (4) definitely plan to leave teaching as soon as I can, and (5) undecided at this time. Similar to some previous studies examining teacher retention using prior iterations of this data set (Sentovich 2004; Stockard and Lehman 2004), this study placed the fifth response option of “undecided at this time” between the second response option of “until I am eligible for retirement” and the third response option of “will probably continue unless something better comes along.” The second question asked the teacher if he or she could go back to his or her college days and start over again, would he or she become a teacher or not. It also had five possible response options, with them being: (1) certainly would become a teacher, (2) probably would become a teacher, (3) chances about even for and against, (4) probably would not become a teacher, and (5) certainly would not become a teacher. These items were both reversely recoded for interpretability.

#### Weighting of the data

The sample in this study was taken from a nationally representative complex sample design that consisted of stratification of the data, clustering (i.e., the selection of teachers within each school), and over-sampling of certain teacher populations, e.g., teachers who were Native American, which ensured that the samples of these teachers were large enough to produce reliable estimates. In data sets compiled using complex sample designs such as this, direct estimates of the sampling errors based on the assumption of simple random sampling will usually underestimate the sampling variability in the statistical analysis of the data and distort tests of statistical significance (Hahs-Vaughn 2005; Thomas and Heck 2001). To produce unbiased population estimates, teacher final sampling weights provided by the National Center for Education Statistics (NCES) were used in the data analysis of the study (NCES 2008). Weights depend on both the sampling plan and the conceptual orientation of the study, so using the teacher-level weights were deemed appropriate for the data analysis in this study, which focuses on teacher-level inferences. Accordingly, employing sampling weights made the results of the data analysis generalizable to the population of the USA’s entire body of secondary math and science teachers in public schools.

#### Factor analyses

For the school related factors, factor analyses were conducted to condense the large number of items to a more manageable number for use as observable indicators for the latent variables. First, items that were clearly not relevant to the study were removed from the data set, thus leaving only the items possibly related to the variables of interest in the study, e.g., administrative support for the teachers. Factor analyses of the remaining items helped discern what underlying factors, or scales, explained the pattern of correlations that existed among the items. Through the factor analyses, it was determined how many scales would be used, how they could be characterized, and how each scale would be comprised of which items. Cronbach’s alpha values for each scale were also calculated to check the reliabilities of the scales. Several guidelines were followed in determining which items to keep in the scales. In general, items with loadings of .40 or above were considered favorable for inclusion in a scale (Hair et al. 1998; Ingersoll 2001). Cronbach’s alpha values of .6 and above for each full scale were considered acceptable for use in modeling.

A descriptive analysis was then conducted on the resultant factors and their items to gain an understanding of their overall data structure, using frequencies, ranges, means, standard deviations, and variances.

The variables in the model for this study ended up being satisfaction with salary, teacher autonomy, years of experience, administrative support, socioeconomic impact of student families, student truancy, and the intentions of math and science teachers to remain in the profession, with a total of 24 items comprising these scales. Satisfaction with salary had two observed indicators in this study, one that was absolute to teachers’ salaries in their field and one that was relative to salaries in other fields. Teacher autonomy had six observed indicators which pertained to various planning and teaching components that the teacher has control over in the classroom. Years of experience had a single observed indicator directly measuring it. Administrative support had five and both the socioeconomic impact of student families and student truancy had four observed indicators measuring math and science teachers’ perceptions of problems within those areas of their schools’ climates. The intentions of math and science teachers to remain in the profession, as the dependent latent variable in the model, had two indicators measuring it.

#### Structural equation modeling

Substantively, the model proposed in this study focused on a number of relationships between variables concerning the intentions of math and science teachers to continue teaching in secondary public schools, and SEM offered the ideal approach to examine them collectively. The need to determine relative variable strength and scrutinize the theoretical relationships simultaneously in the model called for a holistic approach to model testing, whereas more traditional forms of modeling (e.g., multiple regression) would have only provided separate mini-tests of model components that are conducted on an individual basis (Tomarken and Waller 2005). The selection of SEM not only allowed the set of variables to be analyzed much like independent and dependent variables in regression analysis but also provided a more nuanced understanding of the structural relationships proposed in this study.

Following the two-step procedure recommended by Anderson and Gerbing (1988), the SEM approach in this study consisted of two parts: the measurement model and the structural model. The measurement model first specified the relationships between variables, which were unobserved, constructed factors, and their indicators, which were observed variables, that is, questionnaire items comprising those factors. In other words, it showed how the variables were measured in terms of the observed indicators, given the validity and reliability of the observed indicators (Kline 1998). This involved confirmatory factor analysis (CFA) to validate the measurement model before fitting the structural model. The structural model then specified the relationships between variables and detailed the causal effects and amounts of unexplained variances. Each variable had its own measurement equation and was either exogenous (independent) or endogenous (dependent). While exogenous variables served as predictors for other variables in the structural model, endogenous variables acted as outcome variables in the causal relationships. Both measurement and structural models were estimated using the maximum likelihood (ML) method in LISREL, version 8.80.

##### Type of input matrix

In terms of data input, a covariance matrix, as opposed to a correlation matrix, was generated and used in this analysis (see Table 8 in Appendix 2), and this was done for three reasons. First, Hair et al. (1998) recommend that a covariance matrix be used when testing a proposed theoretical framework, as was the case in this study. Second, Bentler et al. (2001) state that SEM was developed behind statistical theory that rested primarily on the assumption a covariance matrix was to be used. Third, the latent variable model in this study had standardized solutions as well unstandardized ones, and a correlation metric is provided despite the input of a covariance matrix.

##### Normality

Normality tests, i.e., univariate and multivariate normality tests, with reference to the values of skewness and kurtosis of the observed variables, were conducted in this study to test the assumption of normality in SEM. Many of the observed indicators in this study were measured using four to 6-point Likert-type scales. Although Likert scales are technically ordinal, it is considered a common and acceptable practice, especially in the social sciences, to treat their measurements as interval (Kinnear and Taylor 1991; Malhotra 1996). As such, they were treated as continuous variables to conduct the tests for normality.

##### Estimation technique

Given the data in this study, ML was selected as the estimation method for this analysis. First of all, ML is the default estimation technique in LISREL and is more widely used than other estimation methods, like generalized least squares and full information maximum likelihood methods (Anderson and Gerbing 1988; Baumgartner and Homburg 1996; Diamantopoulos and Siguaw 2000). Second, ML is robust against violations of the multivariate normality assumption and consistently yields efficient estimation when sample sizes are sufficiently large (Anderson and Gerbing 1988; Diamantopoulos and Siguaw 2000). Alternatively, asymptotically distribution-free (ADF) methods, i.e., methods that make no assumptions on the distribution of the variables, like weighted least squares (WLS), could be used. These methods, however, were not used as they can be problematic with large sample sizes (Diamantopoulos and Siguaw 2000).

In practice, the observed variables may often reveal some significant *p*-values for both kurtosis and skewness, in both univariate and multivariate normality tests, which could suggest a potential violation of normality. Given the assumption of multivariate normality in SEM, this could pose a problem. However, according to Bollen (1989), ML is robust against violations of the multivariate normality assumption with large sample sizes, which may be the case in this analysis. It is also important to note, as Barnes et al. (2001) point out, that data from Likert scales are rarely normally distributed in practice, and that, for all practical purposes, ML remains the best possible method for estimation. Given the distributions for the observed variables in this study not being wildly non-normal and the robustness of ML estimation for large sample sizes, it was decided to not transform the data into normalized scores nor use ADF estimation methods.

##### Model evaluation

In terms of model evaluation, the *χ* ^2^ (chi-square) statistic and a group of descriptive goodness-of-fit indices were used. The chi-square fit index is highly sensitive to sample size and the hypothesized model is likely to be rejected when the sample size is large, even though the discrepancy between the sample and model covariance matrices may be small (Fan et al. 1999; Fan and Wang 1998). For this reason, several widely used descriptive goodness-of-fit indices were used to assess model fit. These included the normed chi-square (*χ* ^2^/*df*) and the root mean square error of approximation (RMSEA), which are relatively independent of sample size. When sampling weights are applied, LISREL 8.80 only provides RMSEA for the model fit index. RMSEA is a parsimonious fit index that evaluates the overall discrepancy in model-to-data fit while also taking into account the model’s simplicity. RMSEA values of .05 or less, as a rule of thumb, were considered as indicating a good fit (Cudeck and Browne 1993; Hoyle 1995). Hu and Bentler (1999) suggested a cutoff of .06 to show good fit. Cudeck and Browne (1993) reported that RMSEA values less than .08 indicate an adequate model fit and less than .05 indicate a good fit. Normed chi-square values less than 5 were considered to be acceptable (Kline 2005). The validity and reliability of all the variables in the model were also assessed using construct validity, error variances, indicator reliability, Cronbach’s alpha (internal consistency), and construct reliability.

## Results

To check for violations of assumptions required for SEM, tests for univariate normality and multicollinearity were performed. In the test of univariate normality for continuous variables using the PRELIS program (Jöreskog and Sörbom 2002), skewness and kurtosis values for each observed variable were inspected. Two of the observed variables in this analysis revealed significant *p* values (*p* > .05) for skewness, and none were significant for kurtosis. With more significant *p*-values for skewness than kurtosis, there was more of a potential problem with the former. This can be problematic because of the normality assumption in SEM. However, according to Bollen (1989), this violation is mitigated when large sample sizes are used, which was the case in this analysis. In terms of kurtosis, absolute kurtosis values of more than 3.0 can affect the fit of the model (Kline 2005). There were no absolute kurtosis values greater than 3.0, suggesting no severe deviations from normality.

To check for multicollinearity problems, the tolerances, variance inflation factors (VIFs), and correlation coefficients were inspected. Tolerance values of less than .1 and VIFs of greater than 10 at the multivariate level could indicate a problem with multicollinearity in the SEM analysis (Kline 2005). The tolerance values ranged from .431 to .930, and the VIFs ranged from 1.075 to 2.322, indicating no problems with multicollinearity at the multivariate level. Correlation estimates of .850 or higher could indicate a problem with bivariate multicollinearity (Kline 2005). Absolute correlation coefficients among observed variables ranged from .007 to .652, indicating no problem with bivariate multicollinearity (see Table 8 in Appendix 2).

In the test of multivariate normality for continuous variables, the measure of relative multivariate kurtosis as calculated by PRELIS equaled 1.097. Being relatively small, this value indicated that, despite there being items without univariate normality, the multivariate distribution of the variables was reasonably normal. Because data from Likert scales are rarely normally distributed in practice (Barnes et al. 2001), an estimation technique equipped to handle such violations, i.e., ML, was employed.

### Evaluation of the measurement model

The measurement model consisted of seven variables, i.e., the socioeconomic impact of student families, student truancy, years of experience, administrative support, teacher autonomy, and the intentions of math and science teachers to remain in the profession, and the 24 items comprising those variables. Table 2 shows what items comprised each variable and their respective pattern coefficients.

**Table 2:** Pattern coefficients for the measurement model

Item	Socioeconomic impact	Student truancy	Years of experience	Administrative support	Teacher autonomy	Satisfaction with salary	Intention to remain
SOCIO1	.78						
SOCIO2	.75						
SOCIO3	.83						
SOCIO4	.63						
TRUANT1		.70					
TRUANT2		.82					
TRUANT3		.82					
TRUANT4		.77					
YEARS			1.00				
ADMIN1				.78			
ADMIN2				.75			
ADMIN3				.70			
ADMIN4				.69			
ADMIN5				.81			
AUTO1					.33		
AUTO2					.37		
AUTO3					.66		
AUTO4					.71		
AUTO5					.50		
AUTO6					.57		
SALARY1						.39	
SALARY2						.78	
INTENT1							.77
INTENT2							.55

*Note. N* = 6588. Pattern coefficients constrained and not estimated in the model = .00 and are presented as blank. SOCIO = socioeconomic impact of student families; TRUANT = student truancy; YEARS = years of experience; ADMIN = administrative support; AUTO = teacher autonomy; SALARY = satisfaction with salary; INTENT = intention to remain in teaching

The disturbances and measurement errors for each variable were assigned a scale using a unit loading index that fixed the residual path coefficient of one of the items to one. SOCIO3, TRUANT3, ADMIN5, AUTO2, SALARY1, and INTENT2 were used as reference indicators in this model. A pair of items in teacher autonomy, i.e., AUTO1 and AUTO2, was allowed to estimate error covariance. In addition, the error variance for years of experience was set to zero (i.e., its standardized factor loading was equal to one).

Overall, the goodness-of-fit indices for the measurement model were evaluated to show a sufficiently reasonable fit, *χ* ^2^ (*df* = 231, *N* = 6588) = 986.42, *p* < 0.001; *χ* ^2^/*df* = 4.27, and RMSEA = .022. As shown in Table 3, the estimated correlations among variables varied widely from -.04 to .87, most of which were statistically significant.

**Table 3:** Correlations among variables for the measurement model

Variable	SOCIO	TRUANT	EXP	ADMIN	AUTO	SALARY	INTENT
SOCIO	–						
TRUANT	.65*	–					
EXP	.07	.10	–				
ADMIN	.33*	.34*	.02	–			
AUTO	.21*	.17*	.10	.34*	–		
SALARY	.30*	.23*	-.04	.37*	.26*	–	
INTENT	.23*	.19*	.10	.37*	.22*	.87*	–

Note. *p < .05, one-tailed. SOCIO = socioeconomic impact of student families; TRUANT = student truancy; YEARS = years of experience; ADMIN = administrative support; AUTO = teacher autonomy; SALARY = satisfaction with salary; INTENT = intention to remain in teaching

### Assessment of validities and reliabilities for constructs and indicators

Once the overall fit of the measurement model was successfully estimated, validities, and reliabilities for constructs (variables) and indicators (items) were assessed. Table 4 shows construct validity, error variance, indicator reliability, Cronbach’s alpha, and construct reliability for all constructs. Construct validity was evaluated by examining the standardized factor loadings, which ranged from .33 (AUTO1) to .83 (SOCIO3) and were all significant at the .05 level. Error variances for indicators ranged from .18 (AUTO3) to .94 (AUTO2).

**Table 4:** Validities and reliabilities for constructs and indicators

Constructs	Indicators	Construct validity	Error variance	Indicator reliability	Cronbach alpha	Construct reliability
Socio-economic impact	SOCIO1	.78	.35	.61	.82	.87
	SOCIO2	.75	.39	.57		
	SOCIO3	.83	.26	.68		
	SOCIO4	.63	.32	.39		
Student truancy	TRUANT1	.70	.54	.48	.86	.88
	TRUANT2	.82	.25	.67		
	TRUANT3	.82	.25	.67		
	TRUANT4	.77	.33	.60		
Experience	YEARS	1.00	.00	1.00		
Administrative support	ADMIN1	.78	.24	.61	.87	.91
	ADMIN2	.75	.29	.56		
	ADMIN3	.70	.32	.50		
	ADMIN4	.69	.38	.48		
	ADMIN5	.81	.23	.66		
Teacher autonomy	AUTO1	.33	1.00	.11	.67	.78
	AUTO2	.37	.94	.14		
	AUTO3	.66	.18	.44		
	AUTO4	.71	.15	.50		
	AUTO5	.50	.37	.25		
	AUTO6	.57	.19	.33		
Satisfaction with salary	SALARY1	.39	.79	.16	.44	.54
	SALARY2	.78	.38	.60		
Intention to remain	INTENT1	.77	.60	.59	.60	.58
	INTENT2	.55	.65	.30		

*Note. N* = 6588. SOCIO = socioeconomic impact of student families; TRUANT = student truancy; YEARS = years of experience; ADMIN = administrative support; AUTO = teacher autonomy; SALARY = satisfaction with salary; INTENT = intention to remain in teaching

Regarding indicator reliability, some of the indicators were reliable, while some had low reliability. It was important that each construct contains at least one, preferably more, reliable indicator (Hair et al. 1998). For example, AUTO1 (selection of textbooks) was the weakest indicator for teacher autonomy (.11), while AUTO4 (grading of students) was the strongest indicator (.55). For the construct of the socioeconomic impact of student families, SOCIO4 (student health) had the lowest reliability of .39, while SOCIO1 (parental involvement) and SOCIO3 (student preparedness) were highly reliable at .61 and .68, respectively. For student truancy, TRUANT1 (problem with tardiness) had the lowest reliability of .48, while TRUANT2 (student tardiness) and TRUANT3 (student absenteeism) were both highly reliable at .67. For administrative support, ADMIN4 (staff recognition) and ADMIN5 (well-run school) were highly reliable at .61 and .66, respectively.

Cronbach’s alpha values ranged from .44 to .87. While a Cronbach’s alpha above .70 is recommended, a coefficient equal to or greater than .60 is widely accepted as an acceptable threshold in the social sciences (Netemeyer et al. 2003). Therefore, those coefficients equal to or greater than .60 were considered to be internally consistent. In general, the fewer items there are in a scale, the lower the internal reliability will be. This was the case for both satisfaction with salary and intention to remain, each with two items comprising their scales. Construct reliability was based on standardized factor loadings, a statistic that measures the amount of scale score variance that is accounted for by all underlying factors. The formula for construct reliability is as follows:

$$ {\left(\Sigma\ \mathrm{standardized}\ \mathrm{factor}\ \mathrm{loadings}\right)}^2/{\left(\Sigma\ \mathrm{standardized}\ \mathrm{factor}\ \mathrm{loadings}\right)}^2 + \varSigma\ \left(\mathrm{error}\ \mathrm{variances}\right). $$

They were all between .54 and .91 and close to the acceptable threshold (Hair et al. 1998).

### Evaluation of the structural model

When the hypothesized model was analyzed with the structural relationships intact and adding an error covariance between AUTO1 and AUTO2 (as in the measurement model), model fit indices determined good fit between the data and the model: *χ* ^2^ (*df* = 236, *N* = 6588) = 1102.88, *p* < 0.001; *χ* ^2^/*df* = 4.67, and RMSEA = .024. All of the path coefficients for the structural relationships were statistically significant at the .05 level (see Table 5). Satisfaction with salary had the largest standardized path coefficient of all the independent variables. In other words, math and science teachers in secondary public schools who had a greater satisfaction with their salary were more likely to have greater intentions to remain in the profession. The socioeconomic impact of student families, student truancy, and years of experience had statistically significant and positive associations with teacher autonomy, although the magnitudes of the latter two were relatively smaller in comparison with the socioeconomic impact of student families. Administrative support and teacher autonomy also had statistically significant and positive associations with the intentions of math and science teachers to remain in the profession, but their magnitudes were relatively smaller than satisfaction with salary.

**Table 5:** Summary of the results of the tests of relative weights

	Teacher autonomy				Intention to remain			
*R* ^2^	.06				.74			
Path coefficients	*B*	*t* value	*p* value	*β*	*B*	*t* value	*p* value	*β*
Socioeconomic impact	.09	4.20	.02	.17*				
Student truancy	.03	1.52	.02	.06*				
Years of experience	<.01	3.27	<.001	.08*				
Administrative support					.04	1.32	.03	.05*
Teacher autonomy					.04	1.04	.04	.03*
Satisfaction with salary					1.18	12.00	.10	.84*

*Note.* 1. *B*: unstandardized path coefficient

2. *β*: standardized path coefficient

3. * *p* < .05

**Fig. 2:**
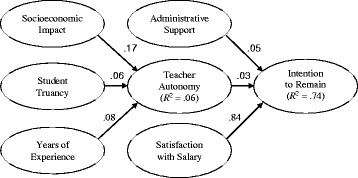
Final structural model

All of the standardized path coefficients were significant at the .05 level. As shown in Fig. 2, the standardized path coefficient from socioeconomic impact to teacher autonomy, which indicates the association of the socioeconomic impact of student families with teacher autonomy when holding the other factors constant, was the largest (*β* = .17) for teacher autonomy. This suggested that the socioeconomic impact of student families associated more strongly with teacher autonomy than student truancy and years of experience. The variances in the three variables together explained 6% of the variance in teacher autonomy (*R* ^2^ = .06). This relatively small proportion of variance associated with teacher autonomy can be attributed to the fact that other variables not accounted for in this study are related to teacher autonomy. The variances in administrative support, teacher autonomy, and satisfaction with salary together explained 74% of the variance in the intentions of math and science teachers to remain in the profession (*R* ^2^ = .74).

The socioeconomic impact of student families, student truancy, and years of experience had a positive correlation with teacher autonomy, partially supporting the left half of the hypothetical model for this study. Administrative support, teacher autonomy, and satisfaction with salary had a positive correlation with the intentions of math and science teachers to remain in the profession, fully supporting the right half of the hypothetical model for this study.

The standardized direct, indirect, and total effects represented by the final structural model were evaluated (see Table 6). The socioeconomic impact of student families and student truancy had a positive direct effect with teacher autonomy (*β* = .17 and *β* = .06, respectively). Contrary to expectations, years of experience also had a positive direct effect with teacher autonomy (*β* = .08). In addition, these three variables had positive indirect effects with the intentions of math and science teachers to remain in the profession (*β* = .005, *β* = .002, and *β* = .002, respectively). Finally, administrative support, teacher autonomy, and satisfaction with salary all had positive direct effects with the intentions of math and science teachers to remain in the profession (*β* = .05, *β* = .03, *β* = .84, respectively).

**Table 6:** Standardized Direct, Indirect, and Total Effects for the Final Structural Model

Dependent variable	Independent variable	Direct effect	*t* value	Indirect effect	*t* value	Total effect	*t* value
Teacher autonomy	Socioeconomic impact	.17	4.20			.17	4.20
	Student truancy	.06	1.52			.06	1.52
	Years of experience	.08	3.27			.08	3.27
Intention to remain	Socioeconomic impact			.005	1.02	.005	1.02
	Student truancy			.002	.83	.002	.83
	Years of experience			.002	.98	.002	.98
	Administrative support	.05	1.32			.05	1.32
	Teacher autonomy	.03	1.04			.03	1.04
	Satisfaction with salary	.84	12.00			.84	12.00

Model comparisons between males and females, whites and minorities, and those in middle and high schools were also made but failed to yield statistically significant differences between groups in each pairwise test.

## Discussion and Conclusion

The retention of math and science teachers in secondary public schools is one of the most critical issues facing education in the USA. It also has a direct impact on the supply of qualified individuals in the associated STEM fields in the USA. Research has shown that nearly half of the US teaching force in these fields leaves the profession within the first 5 years (National Commission on Teaching and America's Future 2003). In order to attract and retain qualified math and science teachers, a number of states and school districts are increasing financial incentives (Hirsch et al. 2001), improving working conditions (Hirsch 2005), and taking steps to raise teacher autonomy through the implementation of professional development programs and/or giving teachers more control over the classroom (Jacob and Lefgren 2004). Determining which of these initiatives is more likely to increase the retention of math and science teachers in the workforce required a better and more focused understanding of the relative importance of these factors on their decisions to remain in or leave the profession.

This study bolstered prior understanding of what keeps math and science teachers in the profession by uniquely combining the following: (1) this study used teachers’ satisfaction with their salaries, as opposed to their actual salaries, since the former is a better reflection of the relative importance of salary to individual teachers, (2) this study incorporated a nationally representative data set to improve generalization of findings to the population of math and science teachers in the USA, (3) with SEM, this study applied the use of a sufficiently sophisticated methodology to this data set, (4) this study examined teachers’ intentions to remain in the profession, as opposed to job satisfaction, since the former presented a better proxy of retention suitable for SEM, and (5) this study focused on math and science teachers in secondary schools, a population of teachers on whom the fields in STEM are so dependent.

The major advantages of SEM over regression modeling in this study were threefold. First, it allowed for simultaneous equation estimation that assessed both measurement issues and causal relationships in a single model and the use of path analysis to statistically and visually illustrate the complex relationships among latent variables in the model (Bollen 1989; Kline 2005). More specifically, it permitted the examination of direct and indirect relationships between multiple independent variables with one or more dependent variables, especially in the instance when there is a locally endogenous variable, i.e., a dependent variable in one equation that becomes an independent variable in another equation (Hair et al. 1998). Teacher autonomy in this study constituted such a case.

Second, SEM accommodated the bias in the estimates due to the measurement error associated with imperfect measures in the data by using multiple indicators for most of the latent variables. As a result, it provided more precise parameter estimates and increased statistical power. Analysis using multiple regression, on the other hand, would have assumed that all constructs are free of measurement error, which is rarely the case in the data found in social sciences (Hox and Bechger 1998).

Third, SEM estimated indirect effects as well as direct effects among latent variables which allowed for the estimation of the total effects. In multiple regression, an indirect effect is commonly overlooked when a hypothesized direct effect is insignificant, so that the variable or relationship is completely dismissed. The path diagram in SEM also helped to clearly present the direction of each effect and the correlations among all variables in one complete picture (Hair et al. 1998; Kline 1998).

For all these reasons, SEM constituted the most fitting way to examine the interrelationships among teachers’ satisfaction with salary, self-efficacy, years of experience, administrative support, the socioeconomic impact of their students’ families, and student truancy that directly or indirectly impacted the intentions of math and science teachers to remain in the profession.

As a result of this study, it is evident that math and science teachers employed at schools with better working conditions, i.e., better administrative support, less student truancy, and more support and involvement from student families with higher socioeconomic backgrounds, were more intent on remaining in the profession. Raising their autonomy also corresponded to a greater intent to continue teaching. Math and science teachers with more years of experience also exhibited an increased likelihood that they would continue with their jobs. Most importantly, math and science teachers’ satisfaction with their salary had the greatest association with their intentions to remain in secondary public schools. Interestingly, the model revealed no statistically significant relationship between satisfaction with salary and teacher autonomy. This suggests that satisfaction with salary, while not influential in terms of their autonomy as teachers, is a particularly important factor in their decisions to remain in the profession. While this study found that satisfaction of salary yielded a strong relationship with math and science teachers’ intentions to remain in the profession, explaining much of the variance in this outcome variable, this variance was further augmented when the administrative support and autonomy of these teachers were also factored in the model.

### Limitations of the study

As comprehensive, nationally representative survey data from SASS08 were used for this study, the limitations of this study were mostly tied to using a secondary data set. Unlike collecting data from a primary source in which the researcher designs the survey to ask specific questions to extract the needed information, this study had to plan the research design and analysis to fit the available data, given the chosen topic. Specifically, SASS08 contained a large number of 4- or 5-point Likert scales that may have limited the degree of the responses. Conducting any follow-up surveys or interviews to further distinguish participant responses was prohibited, as there were strict rules of confidentiality concerning use of this data. Similarly, this research focused on the central elements that were directly part of the teaching job that could be improved by educational policymakers and practitioners, e.g., student truancy, administrative support, and teaching autonomy. External factors that are outside of schools (e.g., alternative job opportunities for math and science teachers) and personal issues (e.g., teachers’ health and family needs) were not discussed in this study. Although these factors are also important to consider when studying the entire issue of teacher retention in secondary public schools, they are generally not controllable by education policymakers and administrators.

While this study covered many variables that are relevant to teacher turnover, there are other variables that were not included in the study due to the data limitations. These variables include teachers’ opportunity for promotion and job security, the quality of professional development, and teachers’ exposure to and attitudes toward high-stakes achievement tests and accountability. In addition, how the variables used in this study, as well as those not considered, might moderate the relationships with math and science teachers’ intentions need to be investigated.

Finally, this study did not account for the effectiveness of the teachers. Ineffective teachers should not be retained, regardless of their intentions. Whereas effective teaching is positively associated with effective school leadership and sustained collaboration between teachers, this study examined a limited aspect of leadership in the form of administrative support, restricted teacher autonomy to individual in-classroom decisions, and tested those factors irrespective of teacher effectiveness. A pronounced focus on what keeps *highly effective* math and science teachers in the profession may yield a more defined plan for outreach.

### Policy implications and considerations for future research

In regards to the socioeconomic impact of student families, it is evident that schools with higher percentages of students who come from families of lower socioeconomic status are more difficult to staff, and teachers tend to leave these schools when presented with alternative job opportunities. By altering policy to make these schools more diverse in this respect, the recruitment and retention of these teachers could be helped. Better student integration and smaller ratios of higher-needs students due to socioeconomic considerations in these schools are examples of policy changes that can help alleviate the attrition of math and science teachers in schools where the socioeconomic impact of student families is detrimental.

Student truancy is another dimension in the retention of math and science teachers that is highly amenable to public policy. By implementing policies that help combat student truancy, its effect on teacher turnover may be mitigated. By instituting policies that dissuade parents from allowing their children to miss school, making efforts to improve student attendance and health, and specifically targeting students who cut class, education policymakers and administrators can aid teachers in becoming more effective in the classroom, thereby raising their job satisfaction and willingness to continue their work.

The experience that teachers accumulate in the profession has a positive bearing on their decisions to remain on the job. By promoting teacher tenure, especially for those who are highly qualified and effective, education policymakers and administrators can help stymie the attrition of math and science teachers in secondary public schools and the effect their attrition has on STEM education. However, increased recruitment of individuals to transition into teaching from other STEM professions may also counter attrition. The experience that they bring into the field was not factored into this study. Differences in training and experience between those who enter teaching directly and those who switch careers need to be examined to determine if alternative routes to teaching are warranted and should be promoted.

It is clear that the support of administrators is crucial to the retention of math and science teachers in secondary public schools. Their communication with teachers about their roles, instructional practice, collaboration with other teachers, and involvement in and implementation of school policies are significant in making the environment in which teachers work more meaningful and desirable. Future research here could focus on what teachers value in terms of administrative support. Math and science teachers, in particular, probably have special needs when it comes to educational resources and technology that could help them bolster their instructional effectiveness. Furthermore, a similar study on principals’ autonomy and their career decisions could be employed to study the effects of the same school factors on the administrators on whom teachers are so dependent. The results of such a study might provide insight into the relationships among various school-level factors, teacher turnover, and principal leadership.

Teacher autonomy is another important facet in the retention of math and science teachers in secondary public schools. Education policymakers and administrators might consider ways to increase the influence of teachers on school policies. For example, they could expand school-based committees that oversee management of the schools and their students to include more teachers. Teacher-based management may be an effective tool for increasing their autonomy as well as school performance. Sharing decision-making power and corresponding responsibility over school policies such as the budget, personnel, disciplinary codes, and curriculum can bring about meaningful change in teaching and learning by investing teachers in all aspects of running a school. Future research could concentrate on how to most meaningfully raise teacher autonomy in this way.

While it has been shown in this study that satisfaction with salary has the strongest association with the intentions of math and science teachers to remain in the profession, this study lacked the views of qualified individuals outside the field of education. The considerations of these individuals were not factored into the study, but they clearly have a bearing on the recruitment of math and science teachers, in particular. Future studies could target this population in formulating a more comprehensive view of how those affiliated with STEM fields make their career decisions and why they choose not to enter the field of education.

By recognizing the significant relationships that the socioeconomic impact of student families, student truancy, years of experience, administrative support, teacher autonomy, and teacher satisfaction with salary have with the intentions of math and science teachers to remain in the profession, education policymakers and practitioners can take measures to increase the retention of these teachers in secondary public schools and help reverse the current trends seen in STEM fields in the USA.

## Appendix 1

**Table 7:** The 2007–2008 SASS items and coding

Construct	Item	Content	Scale	Recoding	Recoded item
YEARS	T0038	How many years have you worked as a full-time elementary or secondary teacher in public schools?	Year(s)		
		How much actual control do you have in your classroom at this school over the following areas of teaching and planning?			
AUTO1	T0280	Selecting textbooks and other instructional materials.	1 = *no control* to 4 = *a great deal of control*		
AUTO2	T0281	Selecting content, topics, and skills to be taught.	1 = *no control* to 4 = *a great deal of control*		
AUTO3	T0282	Selecting teaching techniques.	1 = *no control* to 4 = *a great deal of control*		
AUTO4	T0283	Evaluating and grading students.	1 = *no control* to 4 = *a great deal of control*		
AUTO5	T0284	Disciplining students.	1 = *no control* to 4 = *a great deal of control*		
AUTO6	T0285	Determining the amount of homework to be assigned.	1 = *no control* to 4 = *a great deal of control*		
		To what extent do you agree or disagree with each of the following statements?			
ADMIN1	T0286	The school administration’s behavior toward the staff is supportive and encouraging.	1 = *strongly agree* to 4 = *strongly disagree*	Reverse recode	RE_T0286
SALARY1	T0287	I am satisfied with my teaching salary.	1 = *strongly agree* to 4 = *strongly disagree*	Reverse recode	RE_T0287
ADMIN2	T0292	My principal enforces school rules for student conduct and backs me up when I need it.	1 = *strongly agree* to 4 = *strongly disagree*	Reverse recode	RE_T0292
ADMIN3	T0295	The principal knows what kind of school he or she wants and has communicated it to the staff.	1 = *strongly agree* to 4 = *strongly disagree*	Reverse recode	RE_T0295
ADMIN4	T0297	In this school, staff members are recognized for a job well done.	1 = *strongly agree* to 4 = *strongly disagree*	Reverse recode	RE_T0297
TRUANT1	T0301	The amount of student tardiness and class cutting in this school interferes with my teaching.	1 = *strongly agree* to 4 = *strongly disagree*		
		To what extent is each of the following a problem in this school?			
TRUANT2	T0303	Student tardiness.	1 = *serious problem* to 4 = *not a problem*		
TRUANT3	T0304	Student absenteeism.	1 = *serious problem* to 4 = *not a problem*		
TRUANT4	T0305	Student class cutting.	1 = *serious problem* to 4 = *not a problem*		
SOCIO1	T0309	Lack of parental involvement.	1 = *serious problem* to 4 = *not a problem*		
SOCIO2	T0310	Poverty.	1 = *serious problem* to 4 = *not a problem*		
SOCIO3	T0311	Students come to school unprepared to learn.	1 = *serious problem* to 4 = *not a problem*		
SOCIO4	T0312	Poor student health.	1 = *serious problem* to 4 = *not a problem*		
		To what extent do you agree or disagree with each of the following statements?			
ADMIN5	T0315	I like the way things are run at this school.	1 = *strongly agree* to 4 = *strongly disagree*	Reverse recode	RE_T0315
SALARY2	T0316	If I could get a higher paying job I’d leave teaching as soon as possible.	1 = *strongly agree* to 4 = *strongly disagree*		
INTENT1	T0320	If you could go back to your college days and start over again, would you become a teacher or not?	1 = *certainly would become a teacher*, 2 = *probably would become a teacher*, 3 = *chances about even for and against*, 4 = *probably would not become a teacher*, 5 = *certainly would not become a teacher*	Reverse recode	RE_T0320
INTENT2	T0321	How long do you plan to remain in teaching?	1 = *as long as I am able*, 2 = *until I am eligible for retirement benefits from this job*, 3 = *until I am eligible for retirement benefits from a previous job*, 4 = *until I am eligible for Social Security benefits*, 5 = *until a specific life event occurs (e.g., parenthood, marriage)*, 6 = *until a more desirable job opportunity comes along*, 7 = *definitely plan to leave as soon as I can*, 8 = *undecided at this time*	3 to 2, 4 to 2, 5 to 2, 6 to 4, 7 to 5, 8 to 3, Reverse recode	RE_T0321

*Note.* SOCIO = socioeconomic impact of student families; TRUANT = student truancy; YEARS = years of experience; ADMIN = administrative support; AUTO = teacher autonomy; SALARY = satisfaction with salary; INTENT = intention to remain in teaching

## Appendix 2

**Table 8:** Correlations (*lower left triangle*), variances (*diagonal*), and *c*ovariances (*upper right triangle*)

	1	2	3	4	5	6	7	8	9	10	11	12	13	14	15	16	17	18	19	20	21	22	23	24
1. SOCIO1	*.88*	.53	.55	.31	.32	.30	.39	.32	.46	.14	.15	.11	.18	.23	.14	.11	.06	.04	.13	.03	.13	.16	.17	.12
2. SOCIO2	.56	*.91*	.52	.39	.26	.24	.36	.27	.57	.09	.08	.07	.11	.16	.08	.06	.06	.02	.07	.01	.09	.11	.08	.07
3. SOCIO3	.63	.57	*.81*	.32	.35	.34	.41	.33	.62	.15	.16	.10	.18	.24	.15	.11	.06	.04	.12	.03	.13	.19	.18	.13
4. SOCIO4	.44	.54	.47	*.53*	.20	.20	.26	.22	.17	.07	.07	.07	.09	.13	.05	.01	.04	.03	.06	.04	.08	.10	.06	.04
5. TRUANT1	.33	.24	.38	.28	*1.04*	.53	.49	.50	.63	.15	.18	.11	.15	.20	.08	-.01	.05	.06	.14	.04	.06	.14	.14	.07
6. TRUANT2	.37	.27	.41	.30	.59	*.75*	.50	.50	.65	.13	.15	.09	.13	.19	.07	.02	.03	.02	.09	.00	.06	.10	.10	.07
7. TRUANT3	.47	.40	.49	.39	.55	.65	*.77*	.50	.67	.13	.14	.09	.15	.19	.10	.04	.04	.04	.09	.02	.09	.13	.14	.08
8. TRUANT4	.37	.30	.40	.34	.55	.64	.63	*.82*	.75	.15	.17	.10	.14	.19	.11	.03	.05	.05	.12	.03	.07	.12	.12	.08
9. YEARS	.08	.07	.09	.04	.06	.08	.08	.08	*97.72*	.15	.08	.16	-.03	.14	2.43	.95	.27	.32	.25	.11	-.39	-.27	-1.02	-.38
10. ADMIN	.17	.08	.18	.11	.16	.16	.16	.18	-.01	*.63*	.38	.33	.37	.42	.11	.09	.08	.08	.14	.06	.14	.15	.17	.12
11. ADMIN	.18	.08	.21	.12	.21	.23	.20	.23	.01	.61	*.65*	.37	.34	.40	.08	.06	.06	.07	.16	.05	.10	.14	.21	.13
12. ADMIN	.15	.08	.14	.13	.14	.14	.14	.15	.02	.55	.59	*.63*	.36	.37	.03	.03	.04	.06	.11	.03	.09	.11	.15	.09
13. ADMIN	.20	.11	.21	.12	.16	.16	.18	.16	-.03	.54	.48	.53	*.74*	.40	.06	.03	.06	.06	.12	.04	.14	.16	.23	.14
14. ADMIN	.27	.17	.29	.19	.23	.26	.26	.26	.02	.62	.60	.57	.55	*.69*	.11	.08	.09	.08	.17	.06	.17	.23	.25	.18
15. AUTO1	.12	.06	.15	.07	.10	.10	.11	.13	.24	.09	.08	.03	.04	.09	*1.13*	.50	.15	.12	.09	.09	.08	.07	.09	.04
16. AUTO2	.11	.06	.14	.04	.04	.07	.08	.07	.11	.11	.08	.03	.05	.11	.49	*1.09*	.17	.15	.11	.10	.12	.07	.10	.04
17. AUTO3	.10	.10	.11	.09	.09	.08	.09	.11	.04	.15	.13	.08	.11	.15	.26	.30	*.32*	.14	.12	.12	.06.	.08	.08	.04
18. AUTO4	.07	.04	.08	.06	.07	.07	.07	.11	.05	.14	.14	.08	.10	.14	.22	.27	.46	*.31*	.14	.13	.05	.06	.07	.03
19. AUTO5	.18	.11	.20	.12	.17	.16	.16	.19	.04	.26	.28	.19	.20	.28	.17	.18	.27	.33	*.49*	.10	.08	.11	.13	.08
20. AUTO6	.07	.05	.08	.08	.07	.04	.06	.06	.04	.14	.13	.09	.10	.13	.17	.17	.39	.42	.31	*.28*	.02	.04	.06	.01
21. SALARY1	.12	.07	.12	.08	.07	.07	.10	.08	-.03	.17	.13	.11	.17	.20	.04	.09	.06	.04	.09	.03	*.94*	.29	.28	.16
22. SALARY2	.14	.09	.18	.13	.14	.11	.14	.12	-.01	.20	.18	.15	.19	.26	.05	.06	.12	.08	.15	.09	.28	*.95*	.61	.35
23. INTENT1	.13	.07	.16	.07	.11	.09	.12	.09	-.08	.16	.16	.13	.19	.22	.03	.07	.09	.06	.14	.08	.23	.51	*1.46*	.50
24. INTENT2	.09	.04	.11	.04	.08	.09	.10	.08	.00	.14	.13	.12	.15	.19	.03	.04	.04	.04	.11	.04	.15	.36	.43	*.94*

*Note. N* = 6588. SOCIO = socioeconomic impact of student families; TRUANT = student truancy; YEARS = years of experience; ADMIN = administrative support; AUTO = teacher autonomy; SALARY = satisfaction with salary; INTENT = intention to remain in teaching
